# PIGOME: An Integrated and Comprehensive Multi-omics Database for Pig Functional Genomics Studies

**DOI:** 10.1093/gpbjnl/qzaf016

**Published:** 2025-02-28

**Authors:** Guohao Han, Peng Yang, Yongjin Zhang, Qiaowei Li, Xinhao Fan, Ruipu Chen, Chao Yan, Mu Zeng, Yalan Yang, Zhonglin Tang

**Affiliations:** Kunpeng Institute of Modern Agriculture at Foshan, Agricultural Genomics Institute, Chinese Academy of Agricultural Sciences, Foshan 528225, China; Shenzhen Branch, Guangdong Laboratory for Lingnan Modern Agriculture, Key Laboratory of Livestock and Poultry Multi-omics of MARA, Agricultural Genomics Institute at Shenzhen, Chinese Academy of Agricultural Sciences, Shenzhen 518124, China; GuangXi Engineering Centre for Resource Development of Bama Xiang Pig, Bama 547500, China; Kunpeng Institute of Modern Agriculture at Foshan, Agricultural Genomics Institute, Chinese Academy of Agricultural Sciences, Foshan 528225, China; Shenzhen Branch, Guangdong Laboratory for Lingnan Modern Agriculture, Key Laboratory of Livestock and Poultry Multi-omics of MARA, Agricultural Genomics Institute at Shenzhen, Chinese Academy of Agricultural Sciences, Shenzhen 518124, China; GuangXi Engineering Centre for Resource Development of Bama Xiang Pig, Bama 547500, China; School of Life Sciences, Henan University, Kaifeng 475004, China; Shenzhen Research Institute of Henan University, Shenzhen 518000, China; Shenzhen Branch, Guangdong Laboratory for Lingnan Modern Agriculture, Key Laboratory of Livestock and Poultry Multi-omics of MARA, Agricultural Genomics Institute at Shenzhen, Chinese Academy of Agricultural Sciences, Shenzhen 518124, China; GuangXi Engineering Centre for Resource Development of Bama Xiang Pig, Bama 547500, China; Kunpeng Institute of Modern Agriculture at Foshan, Agricultural Genomics Institute, Chinese Academy of Agricultural Sciences, Foshan 528225, China; Shenzhen Branch, Guangdong Laboratory for Lingnan Modern Agriculture, Key Laboratory of Livestock and Poultry Multi-omics of MARA, Agricultural Genomics Institute at Shenzhen, Chinese Academy of Agricultural Sciences, Shenzhen 518124, China; GuangXi Engineering Centre for Resource Development of Bama Xiang Pig, Bama 547500, China; School of Veterinary Medicine, University College Dublin, Belfield, Dublin, D04 V1W8, Ireland; Kunpeng Institute of Modern Agriculture at Foshan, Agricultural Genomics Institute, Chinese Academy of Agricultural Sciences, Foshan 528225, China; Shenzhen Branch, Guangdong Laboratory for Lingnan Modern Agriculture, Key Laboratory of Livestock and Poultry Multi-omics of MARA, Agricultural Genomics Institute at Shenzhen, Chinese Academy of Agricultural Sciences, Shenzhen 518124, China; GuangXi Engineering Centre for Resource Development of Bama Xiang Pig, Bama 547500, China; Kunpeng Institute of Modern Agriculture at Foshan, Agricultural Genomics Institute, Chinese Academy of Agricultural Sciences, Foshan 528225, China; Shenzhen Branch, Guangdong Laboratory for Lingnan Modern Agriculture, Key Laboratory of Livestock and Poultry Multi-omics of MARA, Agricultural Genomics Institute at Shenzhen, Chinese Academy of Agricultural Sciences, Shenzhen 518124, China; GuangXi Engineering Centre for Resource Development of Bama Xiang Pig, Bama 547500, China; Kunpeng Institute of Modern Agriculture at Foshan, Agricultural Genomics Institute, Chinese Academy of Agricultural Sciences, Foshan 528225, China; Shenzhen Branch, Guangdong Laboratory for Lingnan Modern Agriculture, Key Laboratory of Livestock and Poultry Multi-omics of MARA, Agricultural Genomics Institute at Shenzhen, Chinese Academy of Agricultural Sciences, Shenzhen 518124, China; GuangXi Engineering Centre for Resource Development of Bama Xiang Pig, Bama 547500, China; Kunpeng Institute of Modern Agriculture at Foshan, Agricultural Genomics Institute, Chinese Academy of Agricultural Sciences, Foshan 528225, China; Shenzhen Branch, Guangdong Laboratory for Lingnan Modern Agriculture, Key Laboratory of Livestock and Poultry Multi-omics of MARA, Agricultural Genomics Institute at Shenzhen, Chinese Academy of Agricultural Sciences, Shenzhen 518124, China; GuangXi Engineering Centre for Resource Development of Bama Xiang Pig, Bama 547500, China; Guangdong Provincial Key Laboratory of Animal Molecular Design and Precise Breeding, Key Laboratory of Animal Molecular Design and Precise Breeding of Guangdong Higher Education Institutes, School of Life Science and Engineering, Foshan University, Foshan 528225, China; Kunpeng Institute of Modern Agriculture at Foshan, Agricultural Genomics Institute, Chinese Academy of Agricultural Sciences, Foshan 528225, China; Shenzhen Branch, Guangdong Laboratory for Lingnan Modern Agriculture, Key Laboratory of Livestock and Poultry Multi-omics of MARA, Agricultural Genomics Institute at Shenzhen, Chinese Academy of Agricultural Sciences, Shenzhen 518124, China; GuangXi Engineering Centre for Resource Development of Bama Xiang Pig, Bama 547500, China; Kunpeng Institute of Modern Agriculture at Foshan, Agricultural Genomics Institute, Chinese Academy of Agricultural Sciences, Foshan 528225, China; Shenzhen Branch, Guangdong Laboratory for Lingnan Modern Agriculture, Key Laboratory of Livestock and Poultry Multi-omics of MARA, Agricultural Genomics Institute at Shenzhen, Chinese Academy of Agricultural Sciences, Shenzhen 518124, China; GuangXi Engineering Centre for Resource Development of Bama Xiang Pig, Bama 547500, China; School of Life Sciences, Henan University, Kaifeng 475004, China

**Keywords:** Pig, Multi-omics, Genome, Gene expression, Epigenetics, Database

## Abstract

In addition to being a major source of animal protein, pigs are an important model for studying development and diseases in humans. Over the past two decades, thousands of high-throughput sequencing studies in pigs have been performed using a variety of tissues from different breeds and developmental stages. However, multi-omics databases specifically designed for pig functional genomics research are still limited. Here, we present PIGOME, a user-friendly database of pig multi-omes. PIGOME currently contains seven types of pig omics datasets, including whole-genome sequencing (WGS), RNA sequencing (RNA-seq), microRNA sequencing (miRNA-seq), chromatin immunoprecipitation sequencing (ChIP-seq), assay for transposase-accessible chromatin sequencing (ATAC-seq), bisulfite sequencing (BS-seq), and methylated RNA immunoprecipitation sequencing (MeRIP-seq), from 6901 samples and 392 projects with manually curated metadata, integrated gene annotation, and quantitative trait locus information. Furthermore, various “Explore” and “Browse” functions have been established to provide user-friendly access to omics information. PIGOME implements several tools to visualize genomic variants, gene expression, and epigenetic signals of a given gene in the pig genome, enabling efficient exploration of spatiotemporal gene expression/epigenetic patterns, functions, regulatory mechanisms, and associated economic traits. Collectively, PIGOME provides valuable resources for pig breeding and is helpful for human biomedical research. PIGOME is available at https://pigome.com.

## Graphical Abstract

**Graphical Abstract:**
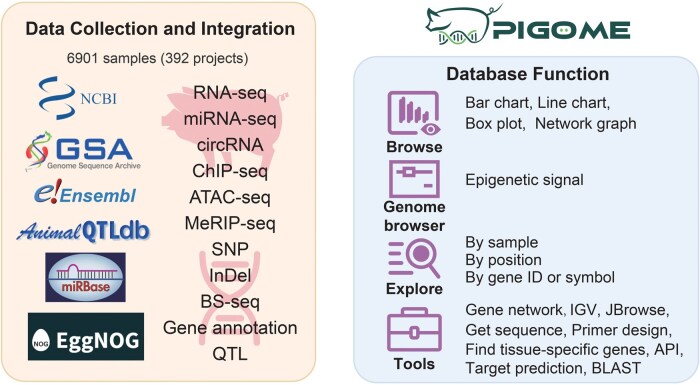

## Introduction

Pig production accounts for a large proportion of the animal husbandry economy and is one of the mainstays of the global agricultural economy [1,2]. Moreover, pigs have been shown to be an important biomedical model for studying human development and diseases [3–5]. Local adaptation and artificial selection have resulted in significant phenotypic differences and genetic diversity in pigs [6], providing a unique opportunity to elucidate the underlying mechanisms of key traits, such as meat production, litter size, coat color, immunity, and diseases [7,8]. Over the past two decades, with the development of advanced sequencing technologies, massive amounts of high-throughput sequencing data have been generated at multi-omics levels. These extensive datasets provide a valuable resource for understanding the genetic mechanisms underlying evolution, selection, trait formation, development, and diseases. They also reveal numerous key variants, genes, and regulatory elements that regulate various biological processes and are associated with economically significant traits [9–11]. Our recent studies, based on high-resolution DNA methylome and transcriptome analyses of skeletal muscle at 27 developmental stages, provided insights into the molecular regulation of skeletal muscle development and diversity. We identified candidate genes, such as insulin-like growth factor 2 mRNA-binding protein 3 (*IGF2BP3*) and SATB homeobox 2 (*SATB2*), that contribute to skeletal muscle development [6,12], offering representative examples of how to integrate multi-omics data to facilitate functional genomics studies in pigs. Therefore, it is necessary to integrate multi-omics data to support scientific discoveries of pig genetics and breeding.

In particular, an increasing number of high-throughput sequencing studies in pigs have been performed on a variety of tissues from different breeds and developmental stages [13–17]. However, these datasets are generated from different laboratories and sequencing platforms, making their retrieval, management, standard processing, and visualization time-consuming and difficult [18]. Furthermore, mining and integrated analysis of these datasets to explore biological functions and regulatory mechanisms remain a challenge [19]. Over the past several years, only a limited number of pig-related databases have been developed. Recently, IAnimal (https://ianimal.pro/) [20] was released, which includes pig multi-omics and genome annotation information. Similarly, ISwine (http://iswine.iomics.pro/) [21] contains published pig genomes, transcriptomes, quantitative traits, and annotation information. The Agricultural Animal Omics Database (AAOD, http://animal.nwsuaf.edu.cn/) contains pan-genome sequencing datasets [22]. However, the analysis and visualization capabilities of these databases are limited (**Table 1**). There is still a lack of comprehensive multi-omics databases dedicated to functional genomics research in pigs.

**Table 1:** Comparison of PIGOME with existing databases

	PIGOME	IAnimal-pig [20]	Iswine [21]	AAOD [22]
Data type	WGS, RNA-seq (mRNAs, lncRNAs, circRNAs, and AS), miRNA-seq, ChIP-seq, ATAC-seq, BS-seq, and MeRIP-seq	WGS, RNA-seq (mRNAs and lncRNAs), ATAC-seq, and ChIP-seq	WGS and RNA-seq (mRNAs and lncRNAs)	WGS
No. of samples	6901	10,714	4107	12
Data volume (Tb)	49.21	132.92	52.88	3.04
No. of breeds	113	65	23	12
No. of tissues	71	132	95	Not provide
No. of developmental stages	29	549	80	Not provide
Analysis tool	JBrowse2, IGV, Get sequence, Primer design, BLAST, Gene network, Target prediction, Find tissue-specific genes, and API	JBrowse, BLAST, Primer design, Gene network, Gene correlation coefficient, Signal plotter, Signal comparison, Genotype plotter, Enrichment, and API	JBrowse, Primer design, BLAST, and Prioritize	GBrowse, BLAST, and BLAT

*Note*: AAOD, Agricultural Animal Omics Database; WGS, whole-genome sequencing; RNA-seq, RNA sequencing; miRNA-seq, microRNA sequencing; ChIP-seq, chromatin immunoprecipitation sequencing; ATAC-seq, assay for transposase-accessible chromatin sequencing; BS-seq, bisulfite sequencing; MeRIP-seq, methylated RNA immunoprecipitation sequencing; mRNA, messenger RNA; lncRNA, long non-coding RNA; circRNA, circular RNA; AS, alternative splicing; IGV, Integrative Genomics Viewer; BLAST, Basic Local Alignment Search Tool; API, Application Programming Interface; BLAT, BLAST-Like Alignment Tool. Data collected until October 2024.

To address these challenges, we developed PIGOME, an integrated and comprehensive web database containing seven types of sequencing data from 6901 datasets and 392 projects, which is currently the most comprehensive omics database for pigs. PIGOME allows researchers to explore and utilize pig multi-omics data easily and effectively. Specifically, PIGOME supports the exploration, analysis, and visualization of genomic variations, gene expression patterns, regulatory networks, and epigenetic modifications for annotated and predicted pig genes [including protein-coding genes (PCGs), long non-coding RNAs (lncRNAs), microRNAs (miRNAs), and circular RNAs (circRNAs)]. PIGOME also includes a tissue-specific analysis tool that allows users to identify gene characteristics in specific tissues. In addition, PIGOME deploys nine tools, such as JBrowse [23], Integrative Genomics Viewer (IGV) [24], and Basic Local Alignment Search Tool (BLAST) [25], to enable users to upload their files to visualize epigenetic signals and perform sequence alignment across the genomes of different pig breeds. In summary, PIGOME is an important database for pig functional genomics studies and will be of interest to a broad readership in the fields of animal genetics, breeding, and biomedical research.

## Data collection and database construction

### Data collection

Seven types of high-throughput sequencing data [whole-genome sequencing (WGS), RNA sequencing (RNA-seq), microRNA sequencing (miRNA-seq), chromatin immunoprecipitation sequencing (ChIP-seq), assay for transposase-accessible chromatin sequencing (ATAC-seq), bisulfite sequencing (BS-seq) for DNA methylation analysis, and methylated RNA immunoprecipitation sequencing (MeRIP-seq)] of pigs were collected from the National Center for Biotechnology Information (NCBI) Sequence Read Archive (SRA, https://www.ncbi.nlm.nih.gov/sra/) and the China National Center for Bioinformation (CNCB) Genome Sequence Archive (GSA, https://ngdc.cncb.ac.cn/gsa/). BS-seq data contain two major types: whole-genome bisulfite sequencing (WGBS) and reduced representation bisulfite sequencing (RRBS) data.

WGS datasets were employed to construct the pan-genome and identify genomic variants, including single nucleotide polymorphisms (SNPs) and insertions and deletions (InDels), within the genome. RNA-seq and miRNA-seq datasets were used to analyze the expression of PCGs and non-coding RNAs (ncRNAs), such as miRNAs, lncRNAs, and circRNAs, and to investigate alternative splicing (AS). ChIP-seq datasets were used to identify the binding sites of CCCTC-binding factor (CTCF), histone modifications, and RNA polymerase II (Pol-II) binding across the genome. ATAC-seq datasets were used to identify open chromatin regions across the genome. BS-seq and MeRIP-seq datasets were used to analyze genome-wide DNA methylation and *N*^6^-methyladenosine (m^6^A) RNA methylation, respectively.

**Figure 1:**
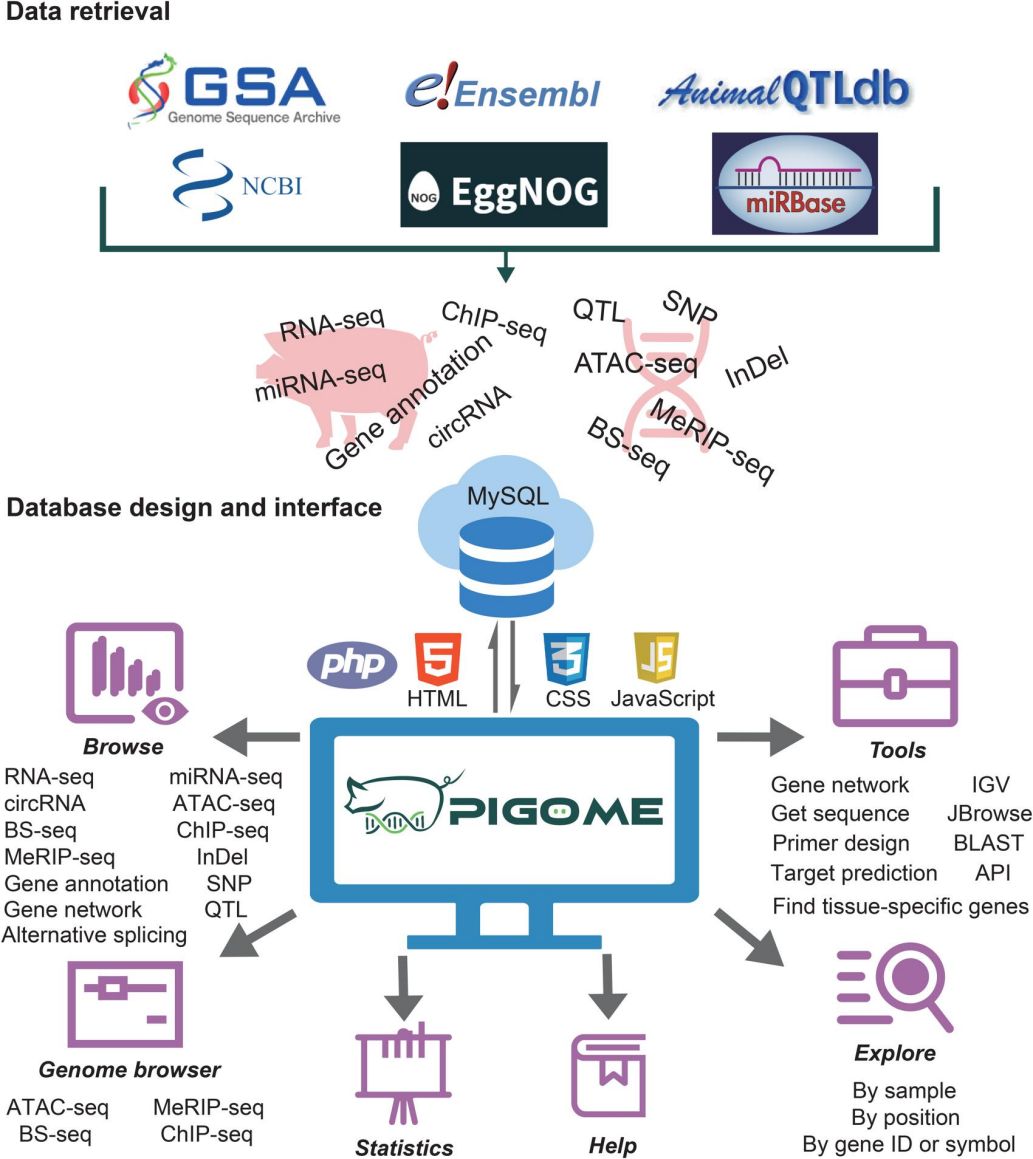
**Database content and construction**The current version of PIGOME integrates seven types of omics data, along with gene annotation, QTL, and alternative splicing information for pigs. PIGOME also provides practical functions and analytical tools for browsing, exploring, and visualizing omics data. NCBI, National Center for Biotechnology Information; GSA, Genome Sequence Archive; Animal QTLdb, Animal QTL Database; QTL, quantitative trait locus; RNA-seq, RNA sequencing; miRNA-seq, microRNA sequencing; ChIP-seq, chromatin immunoprecipitation sequencing; ATAC-seq, assay for transposase-accessible chromatin sequencing; BS-seq, bisulfite sequencing; MeRIP-seq, methylated RNA immunoprecipitation sequencing; circRNA, circular RNA; SNP, single nucleotide polymorphism; InDel, insertion and deletion; HTML, HyperText Markup Language; CSS, Cascading Style Sheets; IGV, Integrative Genomics Viewer; BLAST, Basic Local Alignment Search Tool; API, Application Programming Interface.

All these datasets were manually collected with all the relevant metadata to enable fast and accurate data retrieval and statistical analysis, including project identity (ID), tissue, developmental stage, breed, read number, sequencing platform, reference, and other relevant information. The developmental stages, as described in our previous study [12], were classified into 27 defined stages [embryonic day 33 (E33) to postnatal day 180 (D180)] and two less-defined stages, “Unknown” and “Adult”. Samples with poor data quality (mapping rate < 30% and data volume < 0.15 Gb) were excluded manually. To explore gene functions more conveniently, gene annotation information was integrated from Ensembl 100 [26], miRBase 22.1 [27], and eggNOG 5 [28], while quantitative trait locus (QTL) information was integrated from Animal Quantitative Trait Loci Database (Animal QTLdb 46) [29] (**Figure 1**).

### Data processing

The FASTA format file of the *Sus scrofa* reference genome (build 11.1) and the gene transfer format (GTF) annotation file (release 100) were downloaded from the Ensembl database. All raw FASTQ format files were downloaded from the NCBI SRA database and the China National GeneBank (CNGB) Sequence Archive (CNSA) database. Fastp (v0.20.0) [30] was used to trim and filter the raw reads. For RNA-seq, Hierarchical Indexing for Spliced Alignment of Transcripts 2 (HISAT2, v2.0.5) [31] was used to map the reads to the reference genome. Gene expression in transcripts per kilobase of exon model per million mapped reads (TPM) was calculated using StringTie (v1.3.6) [32]. To analyze AS, each skipped exon (SE) was quantified using the percent-spliced-in (PSI) metric, which was calculated based on the proportions of long and short splice variants detected through replicate Multivariate Analysis of Transcript Splicing (rMATS, v4.0.2) [33]. The PSI matrix utilized in the current database was adopted from our recent study [34]. For miRNA-seq, adapters were removed using Cutadapt (v1.8.dev0) [35]. After adapter removal, reads were aligned to annotated pig miRNAs gathered from miRBase [27] and the pig reference genome using miRDeep2 (v0.1.2) [36]. For circRNAs, HISAT2 (v2.0.5) was used for alignment with the reference genome. Novel circRNAs were identified using CIRIquant (v1.1.1) [37] and find_circ (v2) [38]. For ATAC-seq and ChIP-seq, all reads were mapped using Bowtie 2 (v2.3.5.1) [39]. The peaks were identified using Model-based Analysis of ChIP-Seq 2 (MACS2, v2.2.6) and annotated using SnpEff (v4.2) [40]. Bismark (v0.23.0) [41] was used to align the BS-seq reads to the reference genome using default parameters. All CpG sites were identified and annotated as previously described [12]. For MeRIP-seq, all reads were mapped using HISAT2 (v2.0.5), and peaks were identified using exomePeak2 (v2) [42] and annotated using SnpEff (v4.2). Picard (v2.25.7) was used to remove duplicate polymerase chain reaction (PCR) reads for ATAC-seq, ChIP-seq, and MeRIP-seq. The BigWig files for genome browser visualization were generated using deepTools (v3.5.1) [43] and bedGraphToBigWig (v4). For WGS, all reads were aligned to the reference genome using Burrows-Wheeler Aligner (BWA, v0.7.12) [44]. SNP and InDel calling was performed using the UnifiedGenotyper approach implemented in the Genome Analysis Toolkit (GATK, v4.1.5.0). To achieve high accuracy in variant calling, SNPs and InDels were filtered using the following parameters: QD < 2.0, FS > 60.0, MQ < 40.0, MQRankSum < −12.5, or ReadPosRankSum < −8.0. Considering that the volume of SNP data is too large (∼ 148 GB), SNPs in intergenic regions were not shown on the website, but the raw data could be downloaded from the PIGOME database. Furthermore, tissue-specific genes were identified using the R package TissueEnrich (v3.15) [45] based on the expression matrix of PCGs and ncRNAs. The “rcorr” function of the R Hmisc (v5.1-1) package was used to calculate expression correlations between PCGs, miRNAs, and circRNAs to construct co-expression networks with *r* > 0.85 and *P* < 0.01 as thresholds. The intersection results of RNAhybrid (v2.1.2) [46] and miRanda (v3.3a) [47] were used to predict putative targets for mRNAs and circRNAs with E value < −20.

### Website implementation

PIGOME was built using ThinkPHP (v6.0.12, https://www.thinkphp.cn/), a mature Model–View–Controller (MVC) framework, deployed in CentOS (v7.9) system. All omics data were stored in MySQL (v5.6.50, https://www.mysql.com/). The web interfaces were developed using HyperText Markup Language (HTML), Cascading Style Sheets (CSS), JavaScript, and Bootstrap (v5.0.2, https://getbootstrap.com/). Most of the interactive charts and tables were implemented with ECharts (v5.3.1, https://echarts.apache.org/) and Bootstrap Table (v1.14.2, https://bootstrap-table.com/) (Figure 1). Network proxy services were provided through NGINX (v1.20.1, https://www.nginx.com/). We recommend visiting PIGOME using Google Chrome, Microsoft Edge, or Mozilla Firefox.

**Table 2:** Summary of omics data in PIGOME database

Data type	No. of samples	No. of projects	No. of tissues	No. of breeds	No. of developmental stages	Data volume	Data content
RNA-seq	4217	268	50	74	29	33.92 Tb	31,908 genes
miRNA-seq	995	78	39	32	29	269.35 Gb	544 miRNAs
ATAC-seq	58	5	13	6	5	1.26 Tb	2,884,709 accessible chromatin regions
BS-seq	309	20	26	13	9	2.17 Tb	34,560,764 CpG sites
ChIP-seq	388	16	22	6	7	2.75 Tb	21,318,546 genomic regions
MeRIP-seq	47	5	5	5	10	174.32 Gb	424,376 RNA methylation sites
WGS	887	–	–	53	–	8.67 Tb	12,074,987 SNPs and 5,349,818 InDels

*Note*: miRNA, microRNA; SNP, single nucleotide polymorphism; InDel, insertion and deletion.

## Database content and usage

### Data collection and statistics

Currently, PIGOME v1.0 collects 7 types of multi-omics datasets in pigs, including WGS, RNA-seq, miRNA-seq, ChIP-seq, ATAC-seq, BS-seq (WGBS and RRBS), and MeRIP-seq data. It contains 6901 samples from 392 projects, covering 113 breeds, 71 tissues, and 29 developmental stages. The total clean data volume reaches 49.21 Tb (Table 1 and **Table 2**). The RNA-seq datasets are the most abundant in our database, including 4217 samples, 74 breeds, 50 tissues, and 29 developmental stages (Table 2). To better interpret the omics data, we integrated 32,452 gene annotations, 16,932 SE events, and 29,687 QTLs. Gene annotation records commonly have 22 attributes, including gene symbol, gene type, description, muscle biology, Gene Ontology (GO), Kyoto Encyclopedia of Genes and Genomes (KEGG), Carbohydrate-Active enZYmes Database (CAZy), and Pfam. The AS data include SE events across multiple tissues and skeletal muscle development stages, providing valuable insights into tissue-specific and temporally regulated AS. In addition, the QTL information collects 11 attributes, mainly including position, QTL ID, name, type, trait, and PubMed ID. Additional statistics are summarized on the statistics page (https://pigome.com/statistics.html).

### PIGOME features and functions

PIGOME includes genomic (SNPs, InDels, and genome annotation), epigenomic (chromatin accessibility, histone modifications, and DNA/RNA methylation), and transcriptomic (mRNAs, ncRNAs, and AS) data. In addition, it provides useful and user-friendly tools to help users perform advanced analyzes (Figure 1).

#### Browse

Users can easily browse omics data using a “Browse” tag in the toolbar. After clicking on the omics data type, summary information related to the data will be displayed. On the summary page of each level of omics data, users can obtain specific statistical data, including sample, gene, and other related information, and freely download these tables and charts. For more details, users can click the icon in the “Details” column of a given gene or sample information table on the page, which links to the gene expression page. Basic information about the gene or sample is displayed at the top of the gene expression page, with links to external databases. Different gene expression pages contain different sections. Specifically, the gene expression pages for RNA-seq, miRNA-seq, and circRNA data can show the TPM values in various breeds or developmental stages in given tissues, and also display the TPM values of subgroups of samples freely selected by users. In addition, to better understand the gene function, PIGOME integrates a variety of gene annotations. To better find the co-regulation between genes, the page shows the gene network of the queried gene. The expression pattern of a given gene, as provided by users, can be visualized through box plots, bar charts, and line charts. The data will be conveniently presented in a table below the graph. The AS page enables users to explore the dynamics of SEs across various tissues in Luchuan and Duroc pigs, as well as across 27 developmental stages of the skeletal muscle in Tongcheng pigs. Furthermore, the detail pages for ATAC-seq, BS-seq, ChIP-seq, and MeRIP-seq data provide an IGV genome browser and a table to display information, allowing users to freely explore any genome intervals of each sample. Additionally, users can browse the allele frequency of certain loci in different breeds on the detail page for SNPs and InDels using a bar chart and table. This page also provides QTL information related to this region. In summary, PIGOME has a variety of browsing functions, paving the way for the integration and investigation of different omics data in pigs.

#### Explore

For convenient usage, PIGOME provides three search engines to explore the entire database, including “by gene ID or symbol”, “by position”, and “by sample”. For “by gene ID or symbol”, users can search by entering Ensembl gene ID or official gene symbol. On the “by position” page, users can search by selecting a chromosome and entering the start and end positions. On the results pages for both “by gene ID or symbol” and “by position”, all omics datasets related to a given gene or genomic region are integrated and displayed. Additionally, users can obtain more detailed information by clicking links in the table. For “by sample”, users can fuzzily search by selecting the dataset and entering Sequence Read Archive Run (SRR) ID, sample ID, or project ID. Furthermore, the results page for “by sample” displays relevant sample information with associated links. Importantly, all figures and data from the search results can be downloaded freely and edited easily.

#### Genome browser

PIGOME integrates a custom genome browser based on JBrowse2 to help users compare and analyze various omics datasets. PIGOME contains gene annotation information from Ensembl. By inputting a genome range, gene ID, or gene symbol, users can explore the omics data related to the gene of interest. All tracks are labeled according to the type of omics data, tissues, breeds, and developmental stages. In the track group, users can display tracks of interest by toggling the checkboxes.

#### Tools

We have integrated nine practical tools, including IGV, JBrowse, “Get sequence”, “Primer design”, BLAST, “Gene network”, “Target prediction”, “Find tissue-specific genes”, and Application Programming Interface (API) (Table 1). For IGV and JBrowse, users can check, verify, and interpret their own sequencing and genome data online. The “Get sequence” tool allows users to quickly extract the required gene sequence from a large number of nucleotide sequences. Then, “Primer design” tool [48] can help users design primers from DNA/RNA sequences of interest for further experimental verification. With BLAST, users can perform an alignment analysis using their own sequences against 23 pig reference genomes. The “Gene network” tool is useful for identifying gene regulatory networks formed by the interactions between genes. For “Target prediction” tool, users can explore the regulatory role of a given miRNA in gene expression and find potential functional miRNAs associated with economic traits. Furthermore, a tool called “Find tissue-specific genes” was developed, which helps users quickly find tissue-specific genes, miRNAs, and circRNAs based on our massive RNA-seq and miRNA-seq data. Additionally, for the API tool, users with basic programming skills can obtain the omics data more flexibly and explore functional genes more effectively. These tools will assist users to better explore the biological mechanisms of various biological processes and important economic traits in pigs.

Additionally, users can easily find more help from the database through the “Help tag” in the toolbar.

### Comparison with existing databases

To date, several user-friendly databases have been established to aggregate multi-omics datasets in pigs (Table 1). IAnimal [20] is a multi-species and multi-omics database, encompassing four types of pig omics data, including WGS, RNA-seq (mRNAs and lncRNAs), ChIP-seq, and ATAC-seq data. ISwine [21] serves as a professional pig omics database, offering access to WGS data, RNA-seq (mRNAs and lncRNAs) data, quantitative traits, and annotation information. While IAnimal and ISwine both provide valuable sample meta-information, including details on tissue, developmental stage, and breed, they lack secondary classification and correction of this metadata. Consequently, comparative analysis of gene expression regulation between developmental stages or breeds becomes challenging. The AAOD database [22] focuses on providing 12 *de novo* genome assemblies in pigs. PIGOME, however, emerges as a standout platform in this landscape. Notably, it boasts the widest array of omics data types, including WGS (SNPs and InDels), RNA-seq (mRNAs, lncRNAs, circRNAs, and AS), miRNA-seq, ChIP-seq, ATAC-seq, BS-seq, and MeRIP-seq data (Table 2). Specifically, PIGOME uniquely provides a leading resource for RNA regulation research, offering data on over 500 miRNAs, 150,000 circRNAs, and 16,000 SE events. Furthermore, PIGOME provides meticulously curated and well-organized meta-information on sample data. It supports data from 113 breeds, significantly surpassing other databases and enabling more comprehensive analyses of breed-specific traits and gene regulation. The sample details on developmental stages and tissue organization have been rigorously corrected and refined, ensuring high accuracy. This meticulous curation offers invaluable insights for users aiming to investigate traits with greater precision across diverse breeds, developmental stages, and tissues. Additionally, PIGOME offers nine practical tools designed to enhance the utilization of multi-omics datasets, a feature comparable to that of IAnimal (Table 1). Notably, PIGOME includes useful tools such as “Target prediction”, “Find tissue-specific genes”, “Get sequence”, and “IGV”, which are unavailable in other databases. PIGOME facilitates the functional genomics exploration of tissue-specific PCGs and ncRNAs in a more convenient manner, as shown in the next section.

## Case study

**Figure 2:**
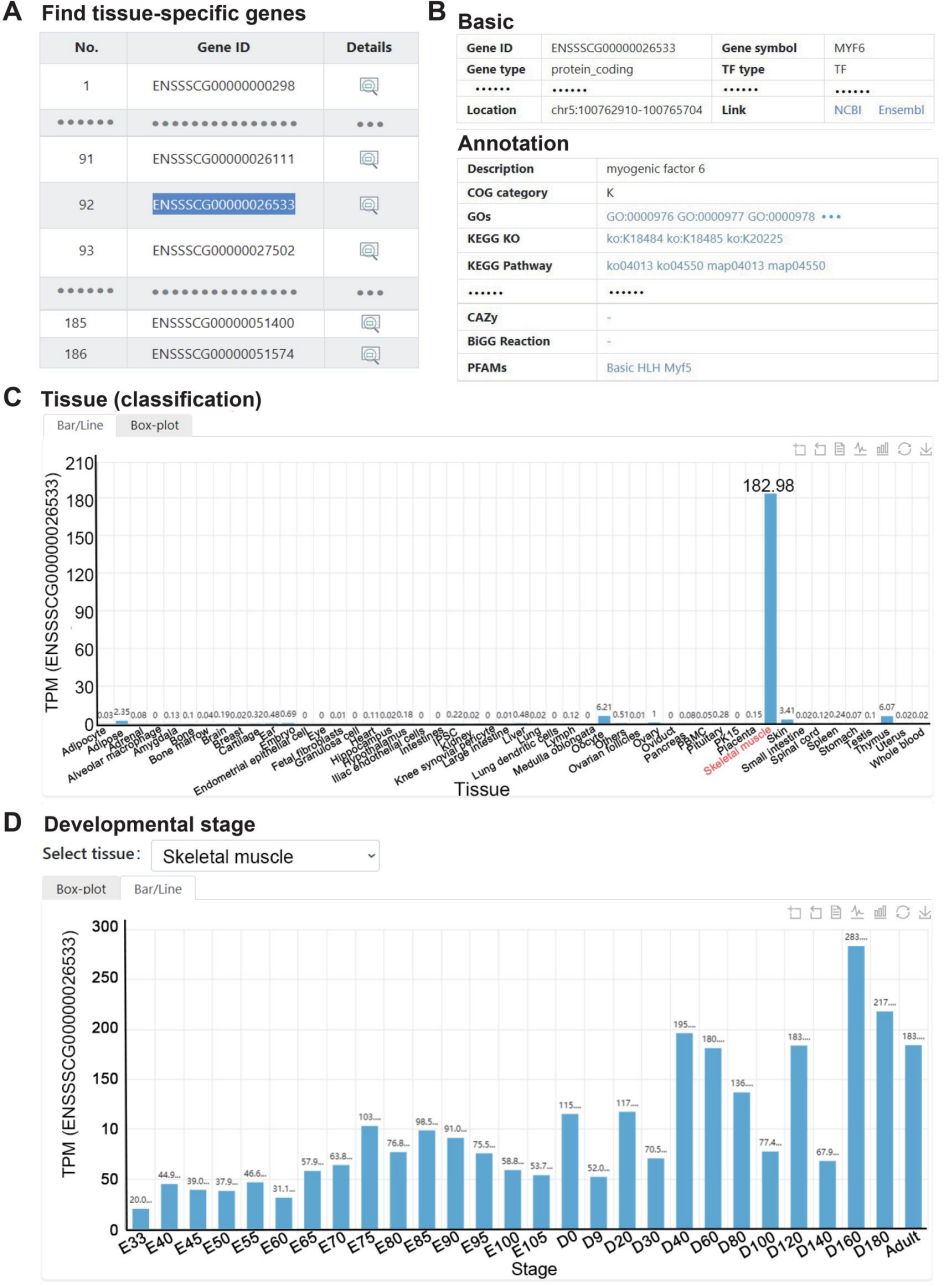
**Find tissue-specific genes module in PIGOME** **A**. The results of finding tissue-specific genes in skeletal muscle. **B**. Basic and annotation information related to ENSSSCG00000026533 (*MYF6*). **C**. The expression of *MYF6* in different tissues. **D**. Expression trend of *MYF6* in skeletal muscle at different developmental stages. TF, transcription factor; COG, cluster of orthologous groups; GO, Gene Ontology; KEGG, Kyoto Encyclopedia of Genes and Genomes; KO, KEGG orthology; CAZy, Carbohydrate-Active enZYmes Database; TPM, transcripts per kilobase of exon model per million mapped reads; E, embryonic day; D, postnatal day.

Herein, we provide a case study to verify the usefulness of the “Find tissue-specific genes” tool in PIGOME and illustrate how to use PIGOME to mine multi-omics information for genes of interest. Initially, users can select the option “skeletal muscle” in the “Find tissue-specifically expressed genes” section of the tool and then click the “Explore” button. On the results page, based on substantial expression data, users can find 186 high-confidence genes that are specifically expressed in skeletal muscle, and can then click the view icon of “ENSSSCG00000026533” to explore more detailed expression information (**Figure 2**A). On the expression page, users can obtain the gene symbol of myogenic factor 6 (*MYF6*), also known as *MRF4*, which encodes a myogenic regulatory factor involved in myogenesis. In addition, users can first view the related gene annotation and visualize its expression in different tissues using bar charts, line charts, or box plots (Figure 2B and C). Importantly, users can also explore the expression trend of this gene in skeletal muscles of different breeds and at different developmental stages (Figure 2D), demonstrating the ability of PIGOME to explore potential tissue-specific genes.

**Figure 3:**
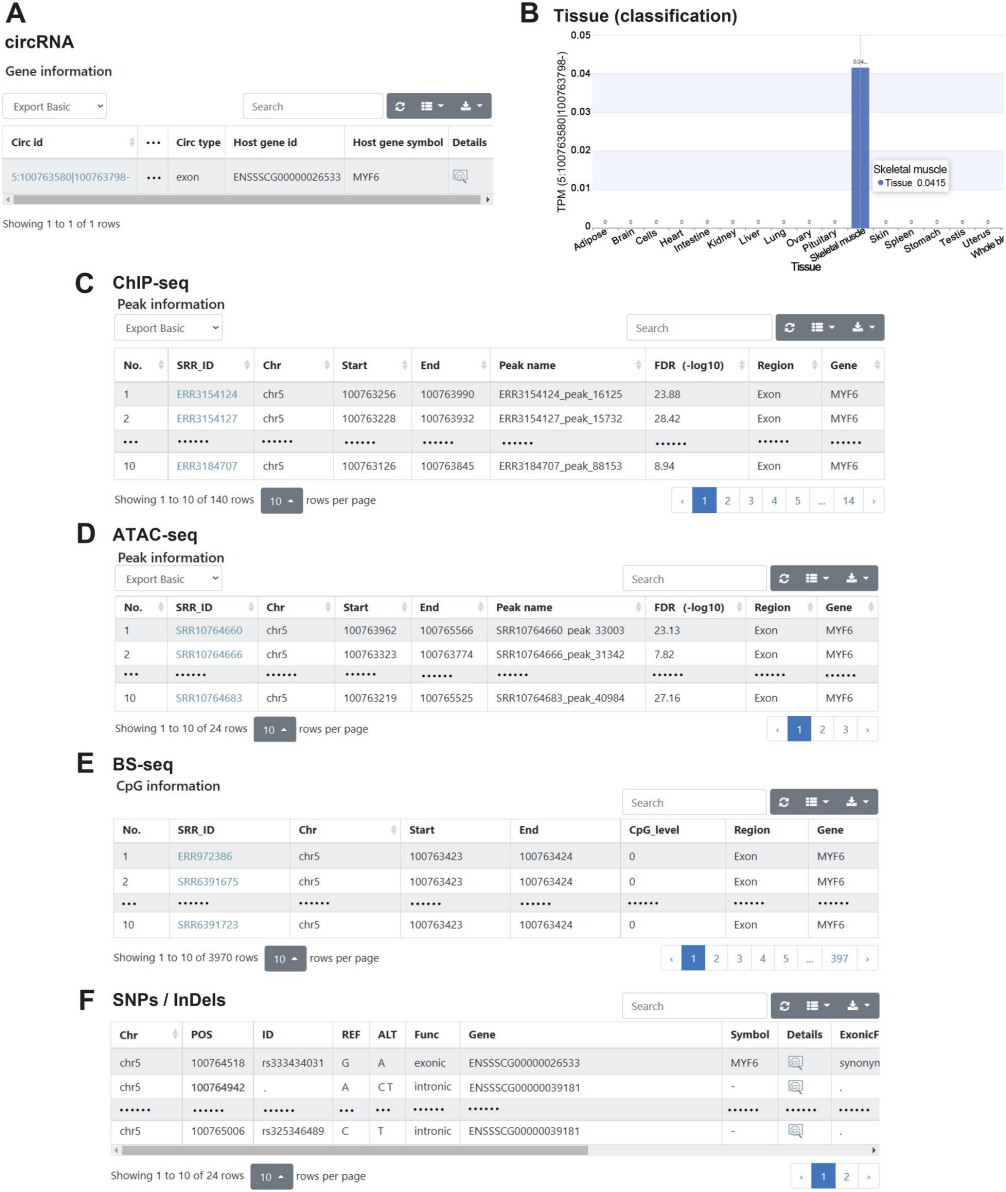
**Exploration of the function and regulation of *MYF6***  **by PIGOMEA**. The circRNA results related to *MYF6*. **B**. Visualization results of *circ-MYF6* expression across different tissues. **C**.–**F**. The ChIP-seq (C), ATAC-seq (D), BS-seq (E), and SNPs/InDels (F) results related to *MYF6*, respectively. SRR, Sequence Read Archive Run; FDR, false discovery rate.

Finally, users can obtain all omics information related to genes using the exploration function. Users can use “ENSSSCG00000026533” or “*MYF6*” as the input in “Explore by gene ID or symbol”. The results page provides information, including SNP and InDel variations, gene expression abundance, AS, annotation, epigenetic modifications, and QTLs related to *MYF6*. More importantly, a circRNA derived from the *MYF6* locus was identified (**Figure 3**A). Interestingly, by clicking the view icon (Figure 3B), data revealed that this circRNA (*circ-MYF6*), which is specifically expressed in skeletal muscle, may be a candidate circRNA that affects the development and growth of skeletal muscle. A total of 140 peaks were identified from the ChIP-seq data, along with ATAC-seq-detected open chromatin regions in the *MYF6* gene (Figure 3C and D). Furthermore, the results showed 3970 CpG methylation sites (Figure 3E) as well as 24 SNPs and InDels (Figure 3F) distributed across the exons, introns, and upstream regions of *MYF6*. These results indicate that PIGOME can be used to explore the potential regulatory mechanisms of candidate genes.

## Discussion

Over the past few decades, researchers have made great efforts in functional genomics research of pigs and have accumulated valuable omics data [49]. Compared to earlier released databases for pigs, such as IAnimal [20], ISwine [21], and AAOD [22], PIGOME contains the most data types and the most up-to-date multi-omics datasets covering comprehensive meta-information (Table 1). Moreover, PIGOME provides a user-friendly interface for browsing and analyzing omics data via interactive webpages, powerful search engines, and advanced tools. The integrated genomic, transcriptomic, and epigenomic data provide an efficient approach for discovering target genes and loci associated with economic traits and human-related diseases.

With the continuous development and innovation of high-throughput sequencing methods, more technologies have been developed, such as single-cell RNA-seq (scRNA-seq), spatial transcriptomics, and 3D genome [50,51]. The amount of omics data in public databases is also increasing rapidly. PIGOME will continue to update new omics types and expand its data volume. In the near future, other types of variations in the pig genome, such as structure variations (SVs), copy number variations (CNVs), and presence/absence variations (PAVs) will be incorporated into PIGOME. We aim to focus on cutting-edge single-cell sequencing and enhance the display of related data, such as scRNA-seq, single-cell ATAC-seq (scATAC-seq), and spatial transcriptomics data. In addition, PIGOME will update the latest gene annotation, genome-wide association studies (GWAS), epigenome-wide association studies (EWAS), and transcriptome-wide association studies (TWAS), and incorporate multi-types of QTL information, such as expression quantitative trait loci (eQTLs) and splicing quantitative trait loci (sQTLs), to help users better understand gene functions. Furthermore, we will strengthen the connections among various data in the database and develop more comprehensive online tools. Finally, we aim to establish PIGOME as a key resource for exploring pig functional genomics, which we believe will be of great value to the broad scientific community in the fields of animal genetics, breeding, and biomedical research.

## Data Availability

PIGOME is available at https://pigome.com. It has also been submitted to Database Commons [52] at the National Genomics Data Center (NGDC), China National Center for Bioinformation (CNCB), which is publicly accessible at https://ngdc.cncb.ac.cn/databasecommons/database/id/9718.

## CRediT author statement

**Guohao Han:** Methodology, Software, Visualization, Writing – original draft. **Peng Yang:** Methodology, Software, Data curation, Formal analysis. **Yongjin Zhang:** Data curation, Formal analysis. **Qiaowei Li:** Formal analysis. **Xinhao Fan:** Formal analysis. **Ruipu Chen:** Formal analysis. **Chao Yan:** Formal analysis. **Mu Zeng:** Formal analysis. **Yalan Yang:** Conceptualization, Project administration, Funding acquisition, Writing – review & editing. **Zhonglin Tang:** Conceptualization, Supervision, Funding acquisition, Writing – review & editing. All authors have read and approved the final manuscript.
